# A simple method for the production of large volume 3D macroporous hydrogels for advanced biotechnological, medical and environmental applications

**DOI:** 10.1038/srep21154

**Published:** 2016-02-17

**Authors:** Irina N. Savina, Ganesh C. Ingavle, Andrew B. Cundy, Sergey V. Mikhalovsky

**Affiliations:** 1 School of Pharmacy and Biomolecular Sciences, University of Brighton, Huxley Building, Lewes Road, Brighton, BN2 4GJ, UK; 2 School of Environment and Technology, University of Brighton, Cockcroft Building, Lewes Road, Brighton, BN2 4GJ, UK; 3 School of Engineering, Nazarbayev University, Astana, 010000, Kazakhstan

## Abstract

The development of bulk, three-dimensional (3D), macroporous polymers with high permeability, large surface area and large volume is highly desirable for a range of applications in the biomedical, biotechnological and environmental areas. The experimental techniques currently used are limited to the production of small size and volume cryogel material. In this work we propose a novel, versatile, simple and reproducible method for the synthesis of large volume porous polymer hydrogels by cryogelation. By controlling the freezing process of the reagent/polymer solution, large-scale 3D macroporous gels with wide interconnected pores (up to 200 *μ*m in diameter) and large accessible surface area have been synthesized. For the first time, macroporous gels (of up to 400 ml bulk volume) with controlled porous structure were manufactured, with potential for scale up to much larger gel dimensions. This method can be used for production of novel 3D multi-component macroporous composite materials with a uniform distribution of embedded particles. The proposed method provides better control of freezing conditions and thus overcomes existing drawbacks limiting production of large gel-based devices and matrices. The proposed method could serve as a new design concept for functional 3D macroporous gels and composites preparation for biomedical, biotechnological and environmental applications.

Macroporous polymer gels have been used in a wide range of applications, including tissue engineering, as cell scaffolds, in bioreactors, materials for biological and chemical separations, and as adsorbents in biomedical and environmental applications. Porosity in gels can be created by different approaches: phase separation^1^ using so-called porogens (chemical additives that generate pores), lyophilization^2^,^3^, via foam formation, and via cryogelation^4^,^5^. The latter method is one of the most versatile techniques used in the last few decades for shaping the porous structure of polymer gels^5^,^6^,^7^. The technology is simple; it commonly requires only one cycle of freezing-defrosting of the reagent/polymer solution, and allows production of materials of varying morphology, mechanical properties and permeability^8^,^9^. The technique is more environmentally friendly than alternative techniques as the most common solvent used is water, and there is no requirement to use organic solvents for removing the pore forming template. The porous polymer is formed in semi-frozen conditions when the major part of the solvent is solidified, forming solvent crystals at temperatures below the normal freezing point, with polymerization taking place in the intercrystalline channels containing unfrozen solution. Increasing the temperature after completion of polymerization leads to defrosting of the solvent crystals and formation of interconnected voids (macropores) in the polymer structure filled with the solvent. Particular interest has arisen due to the high permeability (and consequently low flow resistance) of gels prepared from aqueous solutions and their ability to filter micro- and macro-particles without clogging and pore blockage^10^. This opens the opportunity to design devices for cell and bioparticles separation^10^,^11^,^12^,^13^, direct blood perfusion^14^, tissue engineering^15^,^16^,^17^,^18^,^19^,^20^, water treatment^21^,^22^,^23^ and bioreactors^24^,^25^,^26^. Despite widespread use of the cryogelation technique for macroporous gel production, the phenomenon of freezing of the initial reagent solution and formation of solvent crystals is not fully understood. While some progress has been made on controlling solvent crystal size in small samples by varying the cooling temperature and cooling rate or composition of the reagent mixture, to produce structures with pore gradients and anisotropic pores^27^, there is a recognized difficulty in controlling ice nucleation, and consequently the freezing kinetics and ice crystal formation conditions, in the bulk of larger samples. This leads to poor control of the morphology of the resulting macroporous gels, particularly in larger 3D structures. Indeed, all work performed so far has been done in the laboratory and on a small scale. Gels produced to date are small in size (a few milliliters in volume, or with at least one small (ca. 2 cm or less) dimension), or have variable pore size distribution across the sample. For larger scale separation or engineering/biotechnology applications a simple and reproducible method for the preparation of large volume macroporous gels with improved control of pore structure and permeability is required. Another limitation relates to the manufacture of large macroporous composite materials with nano- and micro-particles embedded in a 3D permeable matrix. The density of the aqueous precursor solutions used for gel formation via the cryogelation method is usually very low, therefore ensuring a uniform distribution of dense nano-particles or micro-particles in gels presents particular difficulty. The particles separate during the freezing process, which results in the failure of the composite entirely or the formation of macroporous gels with uneven particle distribution^8^. This paper studies the freezing of gel systems, and the relationship between the freezing conditions and the morphology of the gel formed. The heat transfer in the bulk of large samples is assessed, and a novel method involving pre-freezing a large volume of the reagent solution prior to commencing gel formation is proposed. For the first time we demonstrate the production of large volume (400 ml or greater) macroporous gel samples and composites with effective and reproducible control of the porous structure. This approach opens new opportunities for producing large volume gels for advanced separation, adsorption or structural applications, and in generating novel 3D structures with embedded micro- and nanoparticles.

## Results

### Conventional synthesis of macroporous gels

**Figure 1:**
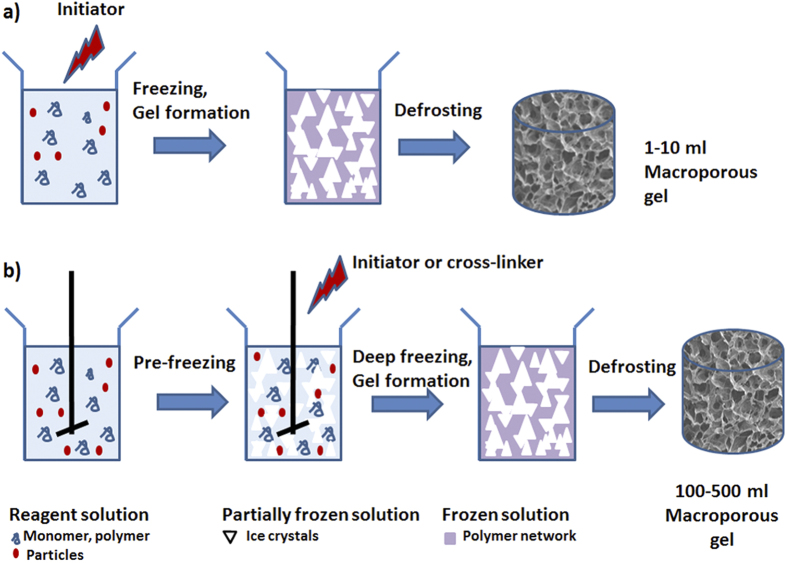
A schematic representation of the method of cryogel preparation by the conventional method (**a**) and the novel (pre-freezing) approach outlined herein (**b**).

Macroporous gels were prepared by cryogelation, involving freezing the initial gel-forming solution and carrying out polymerization or gel formation at temperatures 12–18 degrees below the freezing point of the solvent (Fig. 1a).

**Figure 2:**
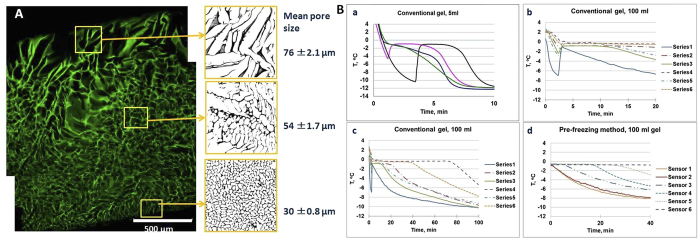
(**A**) Confocal laser scanning microscopy (CLSM) image of a cross section of gelatin hydrogel prepared in a petri dish. The freezing started from the bottom of the petri dish where small pores formed, while larger pores formed at the contact with air (top). The scale bar is 500 mm. (**B**) Temperature profiles measured in HEMA cryogel samples: a) four different samples 5 ml each, b) and c) 100 ml sample prepared using conventional method; and d) 100 ml sample after pre-freezing.

Solvent (ice) crystals formed during the freezing of the solvent act as a porogen. Pores filled with water are formed after defrosting of the material. To obtain a macroporous gel, the solvent crystals need to be formed before the gel forms. To reduce the temperature gradient and also slow down the reaction which leads to the formation of the gel, the reagent solution was pre-cooled in ice before adding the initiator or cross-linker, and also the initiator concentration was reduced to slow down the polymerization itself. The macroporous gel formed after defrosting. The gel morphology depended on the cooling rate and the gel geometry. When a gel was prepared in a Petri dish immersed in a cooling ethanol bath, freezing starts at the bottom of the dish where small ice-crystals quickly form resulting in formation of smaller pores of average size 30 *μ*m (Fig. 2A, bottom). Cooling was slower at the top of the sample leaving more time for ice-crystal growth which results in the formation of larger pores, 76 *μ*m (Fig. 2A, top). The size of the sample was just few (ca. 2) millimeters. It is unsurprising therefore that an increase of the sample size will lead to a bigger temperature gradient in the sample, and large variations in the gel morphology.

### Temperature profiles: Freezing large volumes

The temperature profiles during freezing of 5 ml poly(2-hydroxyethyl methacrylate-polyethylene glycol) (pHEMA) samples with diameter 10 mm at −12 °C were recorded using a temperature sensor inserted in the middle of the sample (Fig. 2B,a). Temperature profiles vary from sample to sample showing the complexity of the freezing process, and its dependence on the nucleation point or the point at which ice crystals begin to grow. Three samples have initially undergone overcooling and remained unfrozen when the temperature was lowered below the normal freezing point of the solvent. Consequently the times at which nucleation and freezing commenced were slightly different. The temperature profiles produced by freezing a 100 ml pHEMA cryogel in a beaker with diameter 120 mm were also recorded (Fig. 2B,b and c). Temperature sensors were inserted in different areas of the sample and show different temperatures depending on their position. The first solution was cooled to 0 °C before the initiator was added. It can be observed that initially the solution was overcooled to −2 to −6 °C. Once crystallization started, the temperature rose back to −0.6 °C. The most rapid temperature changes occurred at the edge of the beaker (Series 1) where the temperature rapidly decreased, while in the middle of the sample cooling and freezing was delayed by 40–70 minutes (Series 4 and 6). As the initiator was added upon initial cooling, the polymerization will start at the same time as the cooling of the sample. Knowing that polymerization is typically complete in about 30 minutes, the delay in the crystal formation has a considerable impact on the gel morphology. The freezing conditions at the sample edge and in the middle of the sample were very different. The nucleation starts at the edge of the sample and its delay in the middle leads to the exclusion and pre-concentration of a larger portion of non-frozen monomer-polymer solution in the center leading to the formation of more elongated pores and thicker polymer walls, and lesser integration. Thus gels are non-uniform in their morphology and are also more fragile.

### Pre-freezing method

**Figure 3:**
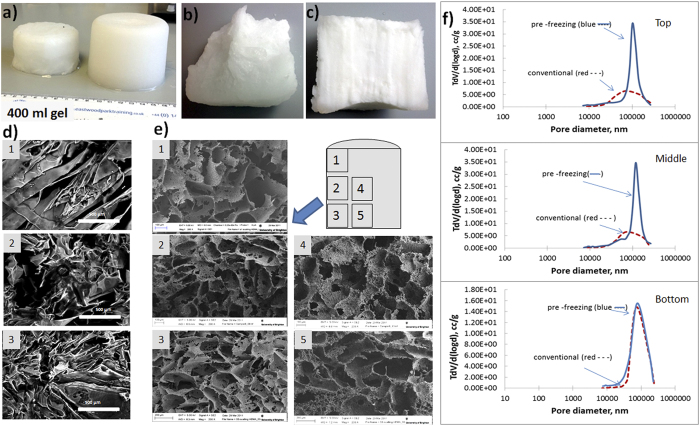
Poly(2-hydroxyethyl methacrylate-polyethylene glycol), pHEMA, cylindrical shape gel (400 ml) produced by the conventional (left) and pre-freezing (right) method (**a**), and its cross-sections (**b**) - conventional, (**c**) - pre-freezing), (**d**) shows cross-sectional confocal laser scanning microscopy images of a conventional gel, while (**e**) shows scanning electron microscopy images of a gel produced by the pre-freezing method, (**f**) shows pore size distribution in three parts of the gels: top, middle and bottom.

To effectively control the freezing of large volumes a partial freezing, or “pre-freezing” of the mixture before initiating gel formation was performed (Fig. 1b). We note that it is possible to partially freeze the solution at temperatures below the solvent freezing point and with constant mixing allow the solvent to crystallize. Between 50 and 90% of the solvent can be frozen out with the gel-forming reagents remaining in the non-frozen liquid phase. Mixing the reaction solution improves the heat transfer and ice nuclei formation. This allows even freezing of large volumes of reaction solution. The temperature profiles of the pre-frozen sample (Fig. 2B,d) are similar to the temperature profiles produced by the conventional method. However in the pre-frozen sample most of the solvent is crystalized and the ice crystals are more evenly distributed within the sample, creating an environment close to a completely frozen block. The initiator was added after solution pre-freezing, thus polymerization was delayed, and occurred in small regions of the non-frozen liquid phase, separated by ice-crystals. The heat transfer in these samples relates to the freezing out of small volumes of the reagent solution and the cooling down of the sample itself. We found that pre-freezing allows production of porous gels of large volume and near-uniform porosity along the whole volume of the sample (Fig. 3). A 400-ml pHEMA cryogel was prepared as one cylindrical piece with dimensions dia. 6 × 10 cm (Fig. 3a). The gel has a macroporous structure along its whole volume with interconnected pores in the size range 20–200 *μ*m (Fig. 3e,f). The conventional gel also has a macroporous structure (Fig. 3), but the pore morphology was different: longer elongated pores formed at the center and top of the sample. The conventional gel is weaker: its compressive modulus is 6 kPa, compared to 12 kPa for the gel made by the pre-freezing method (Table 1). The porosity of the cryogels produced by both methods has been analyzed by mercury porosimetry (Fig. 3f). Three areas in the gels prepared by both methods were selected for analysis: top, middle and bottom. Both types of gel have macropores in the range of 20−200 *μ*m. The pore size distribution was similar for the bottom samples probably due to the similarity of the freezing conditions. The middle and top samples of the gels made by the pre-freezing method however have much narrower pore size distribution compared to the conventional gels. The HEMA gels made by the pre-freezing method also have a larger pore volume compared to the conventional gel (Table 1).

### Dual porosity in pHEMA gels prepared by the pre-freezing method

**Figure 4:**
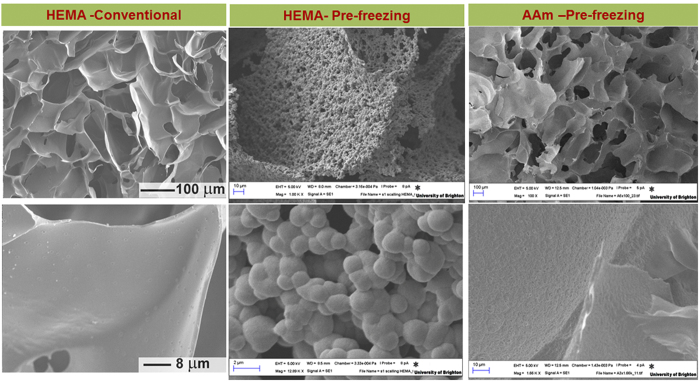
SEM images of a pHEMA cryogel made using the conventional and pre-freezing methods, and a polyacrylamide (pAAm) cryogel made by the pre-freezing method (the morphology of the pAAm gel prepared by the conventional method was similar, data not shown).

The morphology of the pHEMA gel obtained by the pre-freezing method was noticeably different from the one produced by the conventional method (Fig. 4). In the conventional cryogel the gel walls were smooth and no aggregation of the polymer was observed (Fig. 4). During the conventional method small volumes of HEMA solution usually freeze rapidly before polymerization starts, thus polymerization occurs in highly concentrated solutions of small volume with limited diffusion of the polymer. As a result, a polymer with smooth walls is formed. In the pre-freezing method the initiator is added to an already pre-concentrated solution, which leads to rapid polymerization under semi-frozen conditions and subsequent phase separation of the polymer formed in the liquid phase. Additional phase separation between the polymer formed and the solvent resulted in the formation of a more complex structure of polymer aggregates fixed in a 3D structure. SEM images show a unique 3D structure with a combination of macro and micropores in the polymer wall (Fig. 4) and analysis of the porosity shows larger surface area (Table 1). This dual porosity structure is dependent on the thermal and physicochemical characteristics of the monomers used in preparation of the gels: a polyacrylamide (pAAm) cryogel prepared by the pre-freezing method (Fig. 4) showed a similar morphology to samples produced by the conventional method (data not shown).

### Composites

**Figure 5:**
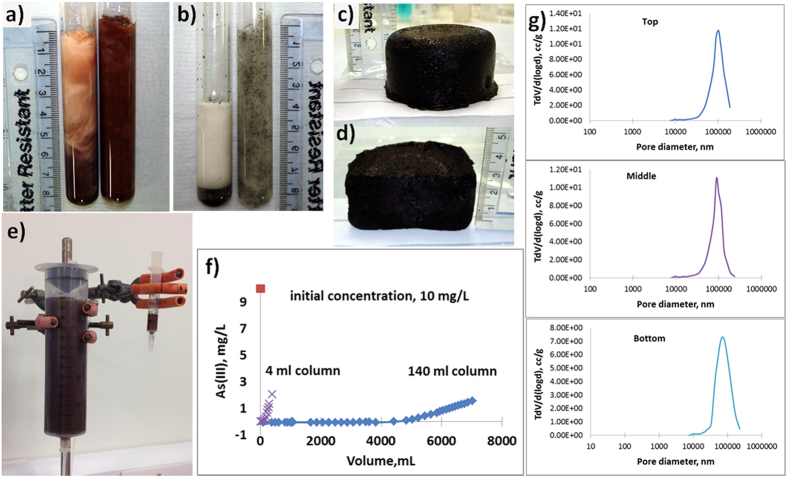
Polyacrylamide cryogel composites. (**a**) with *α*-Fe_2_O_3_ nanoparticles (20 nm) and (**b**) with activated carbon beads (250–500 mm), produced by the conventional method (left test tube) and produced by pre-freezing the suspension before initiation of polymerization (right test tube). The polyacrylamide cryogel composite with carbon nanotubes (**c**) and its cross-section (**d**). *α*-Fe_2_O_3_-AAm column produced by the pre-freezing method (140 ml volume) and conventional method (1 ml volume) (**e**). Adsorption of As(III) by 4 and 140 ml column (**f**). Pore size distribution (**g**). See text for discussion.

Gel-based composites containing iron oxide (*α*-Fe_2_O_3_, 20 nm, Arry International) nanoparticles and microparticles of activated carbon (250–500 *μ*m, MAST Carbon) were prepared by the conventional and pre-freezing methods. The particles were suspended in the reaction solution and divided into two parts. Initiator was added to the first part and the sample placed in a cooling chamber at −18 °C (conventional method). The second part of the suspension was cooled at −18 °C and constantly mixed until the solvent crystallized, forming an ice slurry. After the formation of a thick slurry of ice and particles, the initiator was added and sample was frozen at −18 °C (pre-freezing method). During the pre-freezing method, particle phase separation was inhibited, improving particle distribution across the whole volume of the gel. Figure 5 shows visually that particles were evenly distributed in the sample when the pre-freezing method was used (Fig. 5a,b, right test tube). In a gel prepared by the conventional method the particles of iron oxide and carbon beads tend to settle at the bottom, and form gels with uneven particle distribution (Fig. 5a,b, left test tube). Analysis of iron oxide particle concentration in three samples of a 140 ml cryogel prepared in a 150 ml syringe demonstrates even distribution of the particles through the top, middle and bottom of the gel (Table 1). The particle concentration was about 0.37 ± 0.02 g/g of dried gel. The gel has a pore size distribution between 20 −200 *μ*m (Fig. 5), a pore volume 3.65 ± 0.45 cc/g and surface area 0.198 ± 0.029 m^2^/g (Table 1). Another example is given by a carbon nanotubes-cryogel composite. During composite preparation carbon nanotubes tend to float to the top of the solution and need special procedures for increasing their dispersion in the aqueous solution. The pre-freezing method allows production of large composites with an even distribution of nanotubes, without need for CNT treatment to improve dispersivity (Fig. 5c,d).

### Potential application for water clean-up

A composite column (140 ml) loaded with *α*-Fe_2_O_3_ iron nanoparticles was prepared using the pre-freezing method for through-flow application (Fig. 5e). The column was tested for adsorption of As(III) from aqueous solution and compared with a 4 ml column prepared by the conventional method. The 4 ml column was obtained by stacking together 4 pieces of 1 ml gel. The small pieces of cryogel (1 ml) were prepared to maintain a uniform nanoparticle distribution. A solution of 10 mg/L As(III) was pumped through the column at a 10 ml/min flow rate. The breakthrough profile for the 4 ml column (flow rate 4 ml/min) and the 140 ml column (flow rate 10 ml/min) demonstrates an effective up-scaling of the adsorption process with use of the large column made by the pre-freezing method (Fig. 5f).

## Discussion

**Figure 6:**
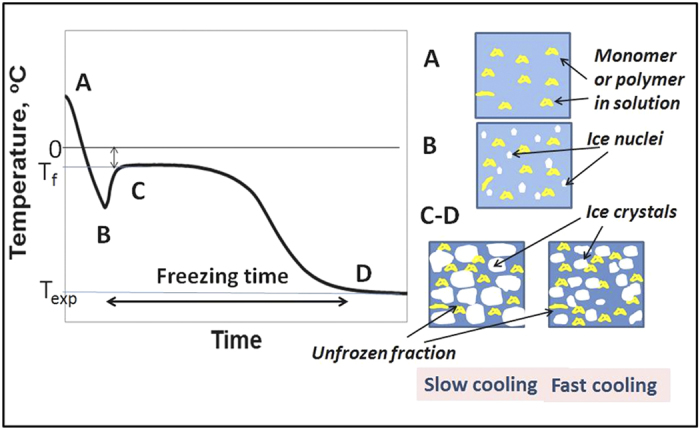
Schematic representation of the freezing process: cooling starts at temperature A, and the solution supercools to B. At B nucleation commences and the temperature rises to C (freezing temperature, T_*f*_). Crystallization and concentration of the solute in the unfrozen liquid continues until D, the target temperature.

The morphology of macroporous gels prepared by cryogelation depends on solvent crystallization, the size of solvent crystals formed, and phase separation between the frozen solvent (ice, when water is used as the solvent) and a reactive gel precursor solution (non-frozen liquid with monomers, polymers, cross-linker, and initiator pre-concentrated within it)^5^,^8^. The freezing rate and freezing temperature influence ice nucleation and growth and thus control the gel porosity^28^. At lower temperatures and fast kinetics of ice-nucleation, gels with smaller pores are formed (Fig. 6). For instance, slush nitrogen is commonly used to freeze samples quickly and prevent any ice crystal growth. However for the synthesis of macroporous materials temperatures in the range of −10 to −25 °C are normally used for aqueous systems to allow larger solvent crystals to grow^7^. The freezing rate is often referred to in the literature as the decrease of temperature with time controlled by the cooling equipment. However it is more important to consider the actual freezing rate of solutions in terms of their transition from liquid to solid and the phase separation which occurs during that time. Practical experiments show that solutions will often cool down to temperatures below freezing point before crystallization commences, known as overcooling (Figs 2 and 6). When the first ice nucleus forms the temperature in the sample will rise to a transition temperature, T_*f*_, which is lower than the freezing point of the pure solvent. The rate of cooling determines the number of nucleation sites and ice crystal size. It is obvious that the sample geometry (i.e. heat and mass transfer dimensions) needs to be considered in this process. Due to the slower freezing rate in the middle of a large sample compared to at its edges, solvent crystallization starts at different times at the sample edge and in the bulk sample (Fig. 2B, b and c). This results in the formation of gels with different morphologies and pore sizes across the sample^8^. Freezing of a 2 mm sample of gelatin leads to a pore distribution varying between 30 and 76 *μ*m (Fig. 2A). Freezing large volumes is even more complicated. As we show in our experiments the time of freezing could vary between 20 and 70 min. This becomes crucial when the polymerization or gel formation is started simultaneously. Thus both processes (freezing and gel formation) occur at the same time and the time for the longer process (solution cooling and ice-crystal formation) becomes the limitation. Freezing of large volumes takes longer than gel formation. Thus at the moment when ice-nucleation starts in the middle of a large sample, the gel is formed. The problem of freezing large volumes uniformly puts severe restrictions on the manufacture of large macroporous gel-based devices. As one solution for making large columns, the approach of stacking together disks produced by freezing samples which are small in one dimension (i.e. as a modular column device) has been suggested^21^. However the technology is laborious and involves additional steps of device construction, which is non-ideal for practical preparation on larger or industrial scales. We suggest here a pre-freezing method with constant mixing of the solution which does not necessarily reduce freezing time, but which facilitates ice nucleation and distribution of ice-crystals in the sample, and delays the start of polymerization or gel formation. In this pre-freezing method the solvent is first allowed to crystallize before the initiator or cross-linking agent is added. The pre- freezing may take anything from minutes to hours depending upon the volume and shape of the mixture concerned. However, as solvent crystals form before the polymerization or gel formation is initiated, the freezing stage does not depend on the gel formation rate. The gel formation is initiated only when the bulk of the solvent is frozen out and so solvent crystals are evenly distributed across the entire sample volume resulting in the formation of large volume gels with better pore size distribution and mechanical properties. Thus pre-freezing presents a simple and elegant solution to the freezing of large samples and production of large-scale 3D macroporous gels, suitable for both large-scale laboratory applications and industrial production. Analysis of the structure of a 400 ml pHEMA gel sample shows the presence of large interconnected pores along the whole sample (Fig. 3). The pore size distribution shows the formation of pores in the range of 20–200 *μ*m. The conventional gel has wider pore size distribution and is weaker and more fragile. Of note is that the pre-freezing of the solution provides conditions for the phase separation of the polymer formed in the non-frozen liquid phase, and formation of novel structures in pHEMA gels. In this work we obtained a dual porosity pHEMA gel, with a complex 3D structure of large macropores (of 20–200 *μ*m) and smaller pores in the polymer wall, making the walls permeable and increasing the polymer surface area. This material has particular interest for biological applications: pHEMA gels are well known biocompatible polymers which have shown good cell adhesion and proliferation characteristics. The gels with added permeability as result of additional porosity in the pore walls may provide better exchange of nutrients and consequent improved cell growth. Such polymer cryogels can be made in the shape of the desired human tissue or organ opening interesting opportunities for their use in regenerative medicine. Macroporous composite materials with an even distribution of particles, which do not easily form a stable suspension, were also prepared by the pre-freezing method. This is valuable in applications where additional reactivity or adsorption/separation functionality is required through the incorporation of non-agglomerated, reactive particles into the polymer walls^21^,^22^,^29^. The mixing and solvent crystals formed at the pre-freezing step prevent the separation separation of the particles from the solution, improving their distribution along the whole sample (Fig. 5). In contrast, the lack of a stable suspension in cryogels prepared by conventional methods results in particle separation in the final composite^21^,^30^. The conventional preparation method is unsuitable for the production of composites where the particles have density which is considerably different from the original solution, or where particles are large in size. For example producing composites with incorporated carbon bead materials or carbon nanotubes results in the formation of gels where the particles concentrate at the top (light particles) or bottom (dense or large particles) of the gel. Increasing the viscosity of the original solution or adding surfactants can help to improve particle dispersion but this is not always desirable, as an increase in viscosity affects the final pore structure, and gels made from a solution with high monomer or polymer content have smaller pores. Further, quick freezing of the samples, in term of reducing the freezing time to limit separation or settling, results in gels with smaller pores. For many compositions it is practically difficult to design the conditions that will stop particle separation. Here we demonstrate that the pre-freezing method is a simple and efficient method for controlling the particle distribution without compromising large pore formation. This finding opens opportunities to develop novel composite materials with large pores and surface area which offer better adsorption and separation performance. Thus we have prepared a *α*-Fe_2_O_3_-AAm composite column of total volume 140 ml. Our previous study shows the potential of using macroporous gels as a scaffold for iron oxide nanoparticles for the generation of reactive nanocomposite devices and their efficiency for As(III) adsorption from aqueous solution. However the application was limited to composites of small size, with a 4 ml column obtained by stacking together 4 separate pieces of the gel tested. Here we upscale the column size to 140 ml and demonstrate effective adsorption at a larger scale. We believe that in industry the column size could be up-scaled further. In conclusion, a novel approach for controlling heat transfer in large volume samples and synthesizing large-scale 3D macroporous hydrogel materials has been developed. Furthermore, the technology can be used to synthesize multi-component, 3D structured composites with improved distribution of particulate material. The proposed method offers the potential to design various sophisticated micro/macroporous, multi-component structures with different size and shape which could have great potential for various applications in the biomedical, biotechnological and environmental sectors.

## Methods

### Gel preparation

Gelatine hydrogel (G gel) sheets were prepared in a glass mould. Glutaraldehyde (3% v/w) was added to the solution of gelatine (6% w/w). The solution was placed in the glass mould and frozen in a Julabo cooling chamber at −12 °C for 20 h. It was then defrosted and washed with an excess of water. 2-hydroxyethyl methacrylate gels were prepared by dissolving 2-hydroxyethyl methacrylate (HEMA, Acros Organic, 98%) and poly(ethylene glycol) diacrylate (PEGDA, Aldrich, Mn ~258) in water (6 w/v% solution, HEMA:PEGDA molar ratio 8:1). The reaction mixture was degassed at low pressure for 25 minutes to eliminate dissolved oxygen before gelation. In the conventional method the mixture was cooled to 0 °C for 15 min and then N,N,N′,N′-tetramethyl-ethylenediamine (TEMED, Fisher Scientific, 99%), and ammonium persulfate (APS, 98%) were added and the mixture allowed to freeze completely. In the pre-freezing method the mixture was cooled with constant mixing in an ethanol cooling bath at −18 °C. After ice crystals formed, the mixture was pre-cooled to −2 °C with constant mixing. N,N,N′,N′-tetramethyl-ethylenediamine (TEMED, Fisher Scientific, 99%), and ammonium persulfate (APS, 98%), were added and the mixture allowed to freeze completely. The frozen mixture was kept at −18 °C for 20 hours and then defrosted at room temperature.

### Composite preparation

Iron oxide nanoparticle composites were prepared by dissolving 4.8 g Acrylamide (AAm, Sigma-Aldrich, 99%) and 1.6 g of N,N′-methylenebisacrylamide (MBAA, Sigma-Aldrich, 96%) in 100 ml of water. The solution was degassed for 20 minutes. Iron oxide nanoparticles (*α*-Fe_2_O_3_, Arry International Group Limited, particle size 30 nm), 10 g and TEMED 0.01 ml were added. 10 ml of the suspension were placed in a glass tube and APS 0.007 g was added. After mixing, the glass tube was placed into a cooling bath at −18 °C. The rest of the suspension of nanoparticles in the monomer solution was pre-cooled in the cooling bath at −18 °C with constant mixing until ice crystals formed. 10 ml of pre-frozen suspension was added to the glass tube and APS, 0.007 g was added. The suspension was mixed and allowed to freeze completely at −18 °C. The frozen samples were kept at −18 °C for 20 hours and then defrosted at room temperature. The iron oxide cryogel of 140 ml was prepared in a similar manner. The reaction solution (150 ml of monomer solution plus 15 g *α*-Fe_2_O_3_) was pre-cooled in the cooling bath at −18 °C with constant mixing until ice crystals formed. The reaction suspension was placed into a 150 ml syringe and initiator was added. The samples were allowed to freeze at −18 °C overnight. The next day the sample was defrosted and washed with water. Activated carbon bead acrylamide composites were similarly made by adding to 100 ml of AAm and MBAA solution carbon beads (MAST Carbon International Ltd, S-250/500-TE9/16–20 °C) pre-wetted in water overnight (3.3 g in 15 ml of water). For making carbon nanotubes composites 6 ml of carbon nanotube suspension (0.09 g of dry weight) was added to 100 ml of AAm (4.8 g) and MBAA (1.6 g) solution. The solution was degassed for 20 minutes and TEMED, 0.01 ml was added. The suspension of nanotubes in the monomer solution was pre-cooled in the cooling bath at −18 °C with constant mixing until ice crystals formed. APS, 0.07 g was then added. The suspension was mixed and allowed to freeze completely at −18 °C. The frozen mixture was kept at −18 °C for 20 hours and then defrosted at room temperature.

### Gel analysis

Hydrogel samples were examined by confocal laser scanning microscopy with a Leica TCS SP5 CLSM using a regular 20x objective lens. A slice of approximately 1 mm in thickness was cut from the wet gel and stained with FITC solution (0.02 mg/ml in sodium phosphate buffer, pH 9.0) for 20 h. The samples were washed out with buffer and water to remove non-bound FITC. The excitation and emission wavelengths used were 488 and 530 nm, respectively. Images were generated by optical sectioning in the xy-planes along the z-axis with 50 optical sections with 1 *μ*m intervals. ImageJ software was used to determine the pore size of hydrogels. For scanning electron microscopy a 400 ml pHEMA gel was cut as shown in Fig. 3(e) and five samples were taken for imaging. Samples were prepared by freeze-drying the gels overnight. After drying, specimens were mounted on aluminum stubs fitted with adhesive carbon pads, sputter coated with platinum and examined using a Zeiss NTS Sigma FEG scanning electron microscope. Cryogels shown in Fig. 4 were prepared and analysed in the same manner. The low and high-pressure mercury intrusion measurements and data analysis were performed using Quantachrome Instruments, USA and PoreWin 6.0 software. Temperature profiles were measured by immersing 6 thermocouples into the reagent solution at different positions. The temperature in the reaction solution during the gel preparation was recorded using a USB TC-08 Thermocouple data logger (Pico Technology) and Easy temperature software. Mechanical properties of the gel were tested using a TA.XTplus Texture Analyser (Stable Micro Systems). The gels were prepared by cutting off 9 mm diameter cylinders from the different parts of the gel. A maximum load of 5 N was applied. The compression modulus at 0.1 strain was calculated using TA.XTplus software. Iron content in the composite cryogels was studied according to^21^. Iron content was measured by extraction of iron with 2 M HCl. Iron concentration in solution was measured using a Perkin Elmer Optima^*TM*^ 2100 DV ICP-OES system.

### Adsorption of As(III)

As(III) solution was prepared according to the method described in^21^. The As(III) solution, 10 mg/L, pH 7.0 was pumped through a 4 ml or 140 ml AAm - *α*-Fe_2_O_3_ composite. A fraction of 10 ml was collected. The concentration of As(III) was measured using a Perkin Elmer Optima^*TM*^ 2100 DV ICP-OES system.

## Additional Information

**How to cite this article**: Savina, I. N. *et al.* A simple method for the production of large volume 3D macroporous hydrogels for advanced biotechnological, medical and environmental applications. *Sci. Rep.* **6**, 21154; doi: 10.1038/srep21154 (2016).

## Figures and Tables

**Table 1:** Gel characteristics.

Gel	Gel section	Pore volume, cc/g	Surface area, m^2^/g	*α*-Fe_2_O_3_ concentration, g/g (dried gel)
*α*-Fe_2_O_3_-AAm 140 ml cryogel	Top	4.08	0.201	0.38 ± 0.01
*α*-Fe_2_O_3_-AAm 140 ml cryogel	Middle	3.18	0.167	0.35 ± 0.01
*α*-Fe_2_O_3_-AAm 140 ml cryogel	Bottom	3.70	0.225	0.37 ± 0.01
Average		3.65 ± 0.45	0.198 ± 0.029	0.37 ± 0.02
**Gel**	**Gel section**	**Pore volume, cc/g**	**Surface area, m^2^/g**	**Compressive modulus, kPa**
HEMA (400 ml) pre-freezing method	Top	8.83	0.453	11.6 ± 0.6
HEMA (400 ml) pre-freezing method	Middle	8.89	0.510	13.4 ± 0.9
HEMA (400 ml) pre-freezing method	Bottom	8.46	0.511	12.0 ± 2.4
Average		8.73 ± 0.23	0.491 ± 0.033	12.3 ± 1.2
HEMA (400 ml) conventional method	Top	5.06	0.331	5.6 ± 0.5
HEMA (400 ml) conventional method	Middle	4.78	0.291	5.7 ± 1.3
HEMA (400 ml) conventional method	Bottom	7.90	0.3388	6.8 ± 2.1
Average		5.92 ± 1.73	0.37 ± 0.049	6.0 ± 1.5
